# The plastid-localized pfkB-type carbohydrate kinases FRUCTOKINASE-LIKE 1 and 2 are essential for growth and development of *Arabidopsis thaliana*

**DOI:** 10.1186/1471-2229-12-102

**Published:** 2012-07-08

**Authors:** Jonathan Gilkerson, Juan Manuel Perez-Ruiz, Joanne Chory, Judy Callis

**Affiliations:** 1 Department of Molecular and Cellular Biology, University of California, 1 Shields Avenue, Davis, CA, 95616, USA; 2 Plant Biology Graduate Group, University of California, Davis, CA, 95616, USA; 3 Plant Biology Laboratory, The Salk Institute for Biological Studies, La Jolla, CA, 92037, USA; 4 Howard Hughes Medical Institute, The Salk Institute for Biological Studies, La Jolla, CA, 92037, USA

**Keywords:** pfkB-type carbohydrate kinase, FLN, Chloroplast transcription, pTAC complex, *Arabidopsis thaliana*

## Abstract

**Background:**

Transcription of plastid-encoded genes requires two different DNA-dependent RNA polymerases, a nuclear-encoded polymerase (NEP) and plastid-encoded polymerase (PEP). Recent studies identified two related pfkB-type carbohydrate kinases, named FRUCTOKINASE-LIKE PROTEIN (FLN1 and FLN2), as components of the thylakoid bound PEP complex in both *Arabidopsis thaliana* and *Sinapis alba* (mustard). Additional work demonstrated that RNAi-mediated reduction in *FLN* expression specifically diminished transcription of PEP-dependent genes.

**Results:**

Here, we report the characterization of *Arabidopsis FLN* knockout alleles to examine the contribution of each gene in plant growth, chloroplast development, and in mediating PEP-dependent transcription. We show that *fln* plants have severe phenotypes with *fln1* resulting in an albino phenotype that is seedling lethal without a source of exogenous carbon. In contrast, *fln2* plants display chlorosis prior to leaf expansion, but exhibit slow greening, remain autotrophic, can grow to maturity, and set viable seed. *fln1 fln2* double mutant analysis reveals haplo-insufficiency, and *fln1 fln2* plants have a similar, but more severe phenotype than either single mutant. Normal plastid development in both light and dark requires the FLNs, but surprisingly skotomorphogenesis is unaffected in *fln* seedlings. Seedlings genetically *fln1-1* with dexamethasone-inducible *FLN1-HA* expression at germination are phenotypically indistinguishable from wild-type. Induction of FLN-HA after 24 hours of germination cannot rescue the mutant phenotype, indicating that the effects of loss of FLN are not always reversible. Examination of chloroplast gene expression in *fln1-1* and *fln2-1* by qRT-PCR reveals that transcripts of PEP-dependent genes were specifically reduced compared to NEP-dependent genes in both single mutants.

**Conclusions:**

Our results demonstrate that each FLN protein contributes to wild type growth, and acting additively are absolutely essential for plant growth and development.

## Background

The plastid, an essential plant organelle, is the sole site of multiple biosynthetic pathways, such as *de novo* fatty acid and amino acid synthesis. After differentiation into the photosynthetically competent chloroplast, membrane-localized light harvesting complexes convert light energy into chemical energy and soluble enzymes reduce CO_2_ into various carbohydrates. Plastid genomes do not encode the complete repertoire of proteins required for these reactions; therefore, nuclear-encoded proteins are present in almost all plastid-localized multi-protein complexes. This requires import of many proteins into the plastid, their targeting to the correct sub-organellar locale, and in most cases, assembly with plastid-encoded subunits. Expression of nuclear-encoded and chloroplast-encoded genes must be coordinated to produce the appropriate subunit stoichiometries and sufficient protein complexes to meet the metabolic needs of the plant [1].

Transcription of plastid-encoded genes is achieved by two different types of DNA-dependent RNA polymerases in land plants [2]. The plastid-encoded RNA polymerase (PEP) resembles bacterial RNA polymerase in sequence identity, requirement for multiple subunits, and use of sigma factors conferring promoter specificity. Plastids contain a second unrelated type, single-subunit nuclear-encoded RNA polymerase (NEP) [3]. Deletions of individual PEP subunit-encoding genes affect the transcription of a specific subset of plastid-encoded genes, indicating the two polymerases transcribe unique sets of plastid genes, although some genes are transcribed by both PEP and NEP [4-6]. PEP can be found in two different complexes, a stromally localized soluble and a membrane attached form [2], the latter referred to as TAC for transcriptionally active chromosome [7]. Interestingly, NEP transcribes the plastid-encoded PEP core polymerase subunits, suggesting a hierarchy of transcription.

Biochemical isolation combined with mass spectrometry of the TAC complex from multiple species [8-12] revealed some additional components, with up to approximately 50 proteins (pTACs) identified in addition to the core PEP polymerase subunits [13]. Of note was the presence of two related proteins in the pkfB-type carbohydrate kinase family, initially reported by Suzuki et al. [12] and confirmed by others [9,14]. These proteins were later named FRUCTOKINASE-LIKE PROTEIN1 (FLN1, At3g54090), and FLN2 (At1g69200) [15]. FLN1 interacts with thioredoxin z and contains 2 vicinal cysteines required for this interaction [15]. The role of the FLN proteins in the PEP complex is unknown and adds yet another dimension to plastid gene transcriptional regulation.

Carbohydrate kinase-like proteins in other organisms have been reported to serve regulatory roles as opposed to a direct catalytic function, while others appear to have both functions. The budding yeast galactokinase-like protein Gal3p lacks catalytic activity but binds both ATP and galactose. The Gal3p-galactose-ATP complex binds to the transcriptional inhibitor Gal80p, reducing Gal80p’s inhibitory effect on the *GAL* promoter [16]. Similarly, a yeast hexokinase 2 isozyme, Hxk2p, an active kinase, has a nuclear signaling function that does not require catalytic activity [17,18]. In *Arabidopsis*HEXOKINASE1 (HXK1) is an active kinase, but similarly is active as a signaling protein even when its catalytic activity is eliminated by mutation [19].

Sugar kinases are grouped into three evolutionarily distinct families unrelated in amino acid sequence and structure – the hexokinase, ribokinase, and galactokinase families [20]. FLN proteins belong to the pfkB-type of carbohydrate kinase, a large group within the ribokinase family, which includes a variety of known and predicted carbohydrate, phospho-carbohydrate and pyrimidine kinases, but remains a poorly characterized group. Members include the two *Arabidopsis* adenosine kinases (ADK) shown to play an important role in adenylate and methyl recycling [21]. Plants with reduced ADK expression have growth defects [22] and reduced root gravitropism [23], defects linked to altered cytokinin levels [24]. In addition to the FLNs, another chloroplast-localized pfkB-type kinase, NARA5, has been characterized and is important for chloroplast development and plastid gene expression [25].

For the two closely related pfkB-type proteins, FLN1 and FLN2, previous work using inducible RNAi in Arabidopsis implied that these two proteins function equivalently in causing chlorosis of emerging leaves [15]. Curiously, using VIGS-mediated expression of RNAi in *N. benthamiana,* the same study showed that reducing tobacco FLN1 induced chlorosis, while reducing FLN2 had very little impact. RNAi’s effects can be variable and often incomplete. Furthermore, *FLN* mRNA was only transiently down-regulated in these experiments. We therefore sought to analyze the effect of FLN loss-of-function in plants genetically null for each individual *FLN*. We analyzed the phenotypes of individual *fln1* and *fln2* null plants, as well as *fln1 fln2* double mutant seedlings. Our results provide new evidence regarding FLN function in *Arabidopsis*. In contrast to the previous results, where in *Arabidopsis* each protein contributes equally, analysis of null alleles reveals a larger role for FLN1. Additionally, we demonstrate for the first time, that together, FLN1 and FLN2 are essential for growth and development. We also show that initial growth in the dark is unaffected in *fln*, supporting the role of FLNs in development of photosynthetic competency. Similar to the previous study, transcription of PEP-dependent chloroplast genes is strongly and specifically affected in each stable *fln* mutant. Finally, using inducible expression, we demonstrate that FLN1 is required early during germination in the light, and expression after cotyledon emergence cannot rescue the mutant phenotype, suggesting a developmentally regulated role for FLNs.

## Results

### *FLN1* and *FLN2* loss-of-function mutants have severe chlorotic phenotypes

**Figure 1:**
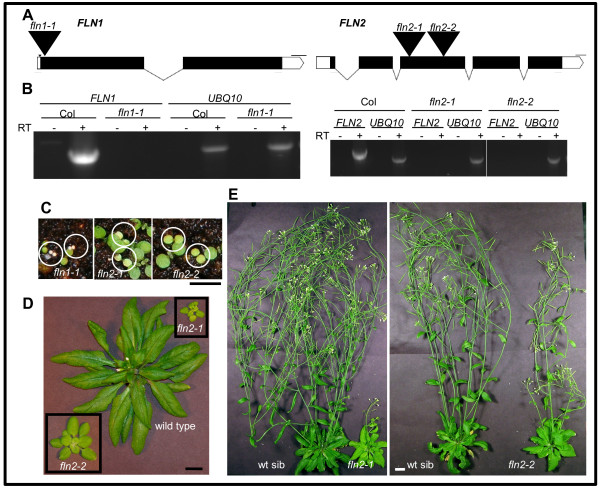
** Characterization of*fln*insertion lines.(A)** Representation of the *FLN1* (At3g54090) and *FLN2* (At1g69200) genes. T-DNA insertions are indicated with large, black triangles (not to scale) with allele designations above them. Exons, UTRs, introns, and deletions are indicated by black boxes, white boxes, lines, and a small triangle, respectively. Scale bars represent 100 bp. Red arrows indicate positions of primers used for RT-PCR. Graphics were generated with Exon-Intron Graphic Maker (http://wormweb.org/exonintron). **(B)** RT-PCR analysis of *fln1* and *fln2* alleles. 7-day old light grown seedlings were used for RNA extraction. The *UBQ10* reactions indicate equivalent RNA inputs for Col-0 and mutant lines, and minus (−) RT reactions are samples treated equivalently except reverse transcriptase was not included in the RT reaction. Experiments are representative of 3 biological replicates. **(C)** 7-day old phenotype of *fln* mutants. Seeds segregating for *fln* alleles grown under a 16 h photoperiod at 18^o^ C. Homozygous individuals are circled. Scale bar represent 0.5 cm. **(D)** 40-day old *fln2* plants. Seedlings from (C) were transferred to individual pots for continued growth. **(E)** 70-day old *fln2* plants. Plant from (D) after 30 more days of growth. For (D) and (E) bars represent 1 cm.

We took a reverse genetics approach and analyzed the available T-DNA insertion alleles to identify the role of each *FLN* in plant growth and development. We analyzed one *FLN1* and two *FLN2* insertion alleles (Figure 1A). Progeny from plants heterozygous for each T-DNA allele were assessed for segregation of any observable phenotypes after germination of seed and subsequent growth on GM plates. Two phenotypes were observed; each line segregated 3:1 for a green:pale/chlorotic phenotype (χ^2^ goodness-of-fit test for 3:1 segregation: *fln1-1*, χ^2^ = 3.5, p-value = 0.060; *fln2-1* χ^2^ = 0.086, p-value = 0.76; *fln2-2*, χ^2^ = 0.030, p-value = 0.86. PCR-based genotyping revealed that the pale/chlorotic seedlings are homozygous for their respective T-DNA insertion.

We confirmed the T-DNA insertion position for each allele with PCR and DNA sequencing across the T-DNA-gene junction (Additional file 1: Figure S1). For *fln1-1* and *fln2-2* only one insertion was detected. PCRs with *fln2-1* T-DNA primers generated a product with either gene-specific primer, suggesting that this allele has two inverted T-DNA insertions (Additional file 1: Figure S1).

To determine if *fln1* and *fln2* T-DNA insertion lines synthesize authentic mRNA, RNA was extracted from pooled pale/chlorotic seedlings and used for subsequent RT-PCR experiments (Figure 1B). Using primers specific to *FLN1*, no product was detectable from *fln1-1* cDNA, whereas a product was visible from Col-0 cDNA (Figure 1B,left panel). Similarly, a product was generated with *FLN2*-specific primers only from Col-0, not from cDNA synthesized from *fln2-1* or *fln2-2* RNA (Figure 1B, right panel). A visible PCR product in all samples using *UBQ10* primers indicated the presence of roughly equivalent input of cDNA template. No product was made when reverse-transcriptase (RT) was omitted from the cDNA synthesis reaction (−RT lanes) indicating that cDNA, not genomic DNA, was the source of template in + RT reactions. These results indicate that the one *FLN1* and the two *FLN2* insertion lines are all likely to be null alleles.

Because the phenotypes originally observed resulted from seedling growth on media supplemented with B-vitamins and 1% sucrose, we sought to test whether the same pale/chlorotic phenotype recapitulated in seedlings grown on soil and to examine if the mutants could grow and develop autotrophically. Seeds from *FLN/fln* parents were sown directly on soil and grown under continuous light (116 μmol/sec^-1^m^-2^). The similar phenotypes observed on GM media were also observed in seedlings grown on soil (Figure 1C). *fln1-1* seedlings have small yellow cotyledons that never green, they do not produce true leaves and die within two weeks after germination. *fln2* mutants grow similarly on soil and on GM plates. They have pale green cotyledons with the cotyledons of *fln2-2* slightly larger than those of *fln2-1* (Figure 1C). The rosettes of the *fln2* alleles are smaller than wild-type, and true leaves slowly green (Figure 1D). *fln2-2* plants have slightly larger rosettes and are greener than *fln2-1*, likely due to the mixed ecotype background of *fln2-2*. Eventually, all *fln2* mutants bolt and make seeds. Adult *fln2-1* plants have fewer inflorescences than do their wild-type siblings, with chlorotic stems, cauline leaves and sepals (Figure 1E). *fln2-2* plants, on the other hand, produce a taller inflorescence compared to *fln2-1*.

### Inducible expression of FLN1-HA complements *fln1-1*

**Figure 2:**
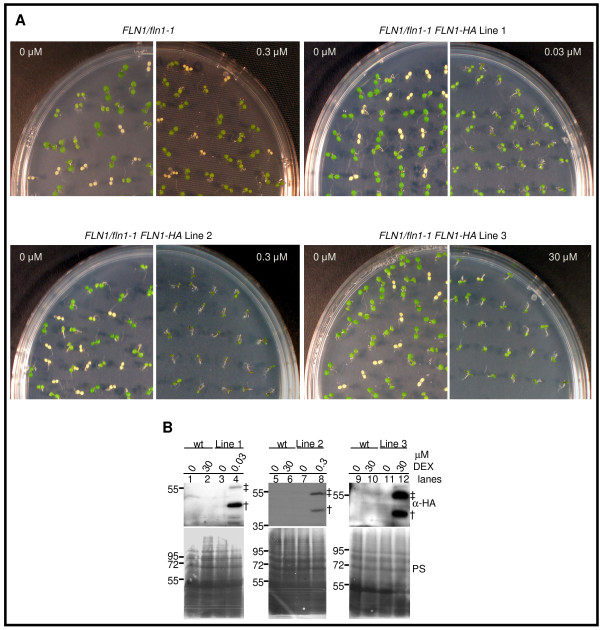
** Complementation of*fln1-1*with dexamethasone-inducible*FLN1-HA*.(A)** Complementation of *fln1-1* phenotype. BC_1_ F_2_ seeds from *FLN1/fln1-1* or T_3_*FLN1/fln1-1 FLN1-HA* seeds were plated on the indicated concentration of dexamethasone and grown for 7 days at 20^o^ C under constant light. 0 μM is DMSO solvent control. Plates shown are one representative experiment of at least three independent experiments. **(B)** FLN1-HA induction by dexamethasone in *FLN1/fln1-1 FLN1-HA* lines. Proteins were extracted from seedlings after 7 days growth, under constant light at 20^o^ C, on GM plates supplemented with indicated concentration of dexamethasone (DEX) and immunoblotted with anti-HA antibodies. Ponceau S stained (PS) membrane is shown as a loading control. Wild-type (wt) plants were green seedlings from a *FLN1/fln1-1* parent without the *FLN1-HA* transgene. Seedlings processed for the “0” treatment for these complementation lines were green siblings (mixed genotype) that segregated with the *fln1-1* phenotype in the absence of DEX. Seedlings for other treatments consisted of a pool of all seedlings (all possible genotypes) on the plate (as *fln1-1* phenotype was complemented on DEX). The presumed FLN1-HA precursor band is denoted by a double-cross, and the mature, processed FLN-HA by a single-cross. Size markers, in kDa, are to the left of blots. Lines 1, 2, 3 refer to three independent *FLN/fln1-1 FLN1-HA* lines. Seedlings were F_3_ seedlings from a *FLN/fln1-1 FLN1-HA* parental genotype.

The similar phenotypes from the two independent insertion alleles of *FLN2* strongly indicate that the mutant phenotype results from loss of *FLN2* expression. However, only one insertion allele of *FLN1* is available. To demonstrate that absence of FLN1 is responsible for the observed phenotype, *FLN1/fln1-1* plants were transformed with expression constructs for constitutive expression of an HA-tagged form of FLN1. All of these transformants exhibited phenotypes consistent with transgene silencing (data not shown), preventing analysis of complementation. To overcome this problem, *FLN1/fln1-1* plants were transformed with a dexamethasone (DEX)-inducible [26,27] version of the same *FLN1-HA* open reading frame. Progeny from three independent *FLN1/fln1-1 FLN1-HA* lines were assessed for phenotypic changes and FLN1-HA induction after growth on DEX-containing media (Figure 2 and Table 1). For all three lines, two anti-HA immunoreactive species could be detected in extracts from seedlings grown on DEX-containing media (Figure 2B, lanes 4, 8, 12), but not in seedlings from the same lines grown on solvent control (Figure 2B, lanes 3, 7, 11) despite loading similar amounts of total protein (Figure 2B, lower panels). This indicates that FLN1-HA expression is DEX-dependent. No anti-HA immunoreactivity could be detected in protein extracts from wild-type, non-transformed control seedlings (green progeny of *FLN1/fln1-1* only) grown either on solvent control (Figure 2B, lanes 1, 5, 9) or on 30 μM DEX (Figure 2B, lanes 2, 6, 10) demonstrating that the protein induced by DEX in the *FLN1/fln1-1 FLN1-HA* lines requires the *FLN1-HA* transgene rather than DEX treatment alone. We presume the 55 kDa species (double-cross, Figure 2B) to be full-length FLN1-HA prior to import into the chloroplast, and the faster migrating species (single-cross, Figure 2B) to be FLN1-HA after import and transit peptide cleavage.

**Table 1:** **Goodness-of-fit test for*fln1-1*segregation in 7-day old T**_**3**_**populations from*FLN1/fln1-1 FLN1-HA*complementation lines grown on the indicated dexamethasone concentration**

**parental genotype**	**[DEX] (μM)**	**green**	**white**	**variegated**	**χ**^**2**^**value**	**p-value**
*FLN1/fln1-1*	0	64	26	0	0.72	0.39
	0.3	88	26	0	0.29	0.58
	30	90	22	0	1.71	0.19
*FLN1/fln1-1 FLN1-HA* Line 1	0	90	27	0	0.23	0.63
	0.03	117	0	0	39.00	<0.0001
*FLN1/fln1-1 FLN1-HA* Line 2	0	85	27	0	0.05	0.82
	0.3	96	0	0	32.00	<0.0001
*FLN1/fln1-1 FLN1-HA* Line 3	0	84	35	0	1.23	0.26
	3.0	95	0	16	6.63	0.01
	30	118	0	6	21.73	<0.0001

Complementation of the *fln1-1* phenotype by FLN1-HA was assessed by observing the segregation ratio of green to white seedlings in progeny from *FLN1/fln1-1 FLN1-HA* parents after germination on DEX media. In *FLN1/fln1-1* progeny lacking the *FLN1-HA* transgene, DEX treatment did not change the 3:1 segregation ratio of green to white seedlings, nor affect the phenotype (Figure 2A, Table 1). However, growth on DEX media abolished segregation of any observable *fln1-1* seedlings in selfed progeny from the three independent *FLN1/fln1-1 FLN1-HA* parents. This result, combined with the tight induction of FLN1-HA by DEX in these same lines (Figure 2B), demonstrates that *fln1-1* can be rescued by DEX-induced expression of FLN1-HA. These lines exhibited complementation on different concentrations of DEX ranging from 0.03 to 30 μM. Lines 1 and 2 fully complement the *fln1-1* phenotype when grown on 0.03 and 0.3 μM DEX, respectively, although line 2 seedlings are smaller (Figure 2A, Table 1). Line 3 partially complements this phenotype when grown on both 3.0 and 30 μM DEX. No white *fln1-1* seedlings were observed either concentration, but several seedlings exhibiting white-green variegated cotyledons were observed at the highest DEX dose used (Figure 2A, Table 1).

**Figure 3:**
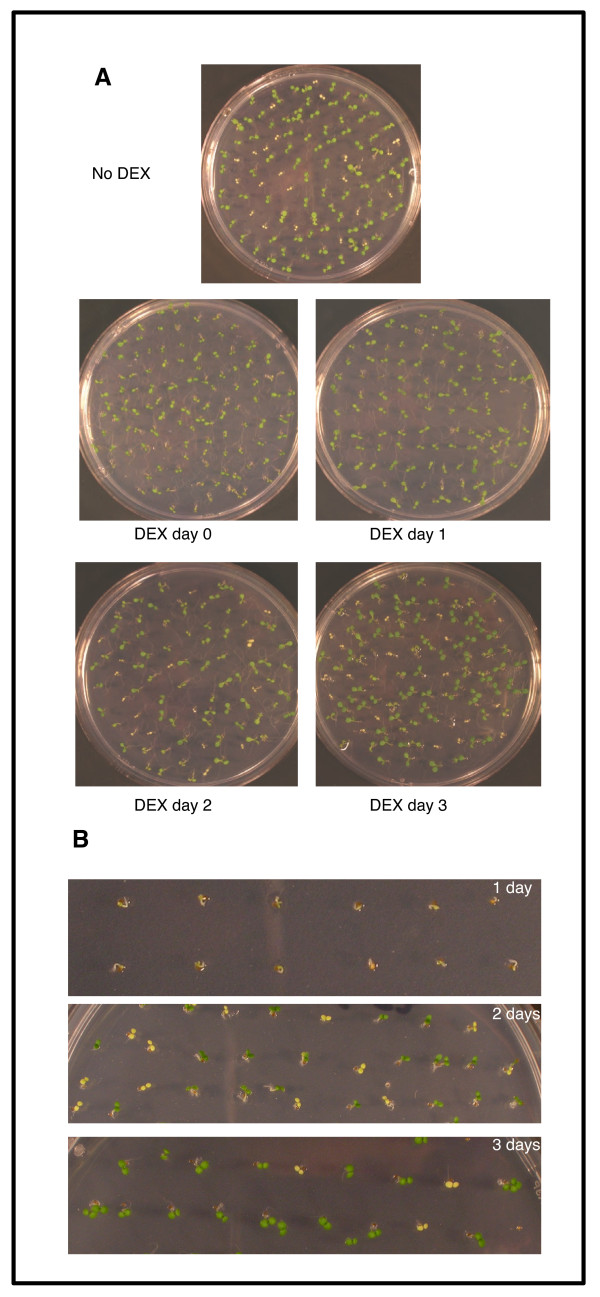
** Time-dependency of*fln1*complementation with DEX treatment.(A)** Seeds from *FLN1/fln1-1 FLN1-HA* line 2 were plated, stratified for 2 days at 4 °C, then incubated at RT. DEX was added at the indicated time after incubation at RT and photographed at 7 days. **(B)** Photographs of seedlings at 1, 2 and 3 days after germination immediately prior to DEX addition, showing the extent of seedling growth at time of DEX application. The experiment in (A) was performed 3 times with 3 independent lines and results were similar for all lines and replicas. Representative pictures are shown.

The ability of FLN1-HA to rescue the mutant phenotype at various times after germination was investigated to assess how early FLN1 expression is required for wild type growth. Seeds from *FLN1/fln1-1* FLN1-HA lines were plated, stratified for 2 days at 4 °C and then grown at room temperature (RT). DEX was added to the plates when first moved to RT (0 day), or 1, 2 or 3 days later. The number of pale seedlings was scored after 7 days (Figure 3). DEX addition immediately after stratification (0 day) and after 1 day rescued the mutant phenotype, as no or only a few pale seedlings were observed (Figure 3A, Table 2). However, DEX addition after 2 or 3 days of growth failed to rescue all three lines because pale seedlings were observed at the expected ratio (Figure 3A, Table 2).

**Table 2:** Dexamethasone induction of FLN1-HA rescues the mutant phenotype only when applied by 24 hours after plating

**parental genotype**	**time after dex (days**)	**green**	**white**	**χ**^**2**^**for 3:1 green:white**	**p-value**
*FLN1/fln1-1 FLN1-HA* Line 2	0	108	0	36	<0.0001
	1	206	0	68.667	<0.0001
	2	156	41	1.843	0.1746
	3	173	41	3.894	0.0485
*FLN1/fln1-1 FLN1-HA* Line 3	0	112	7	23.2	<0.0001
	1	203	19	32.0	<0.0001
	2	169	55	0.024	0.8774
	3	163	56	0.038	0.8453

To determine the developmental stage at which FLN1-HA expression is able to restore *fln1* seedlings to wild type, the development of the seedlings was noted immediately prior to DEX addition (Figure 3B). After 1 day, radicle emergence was observed, but cotyledon expansion had not yet occurred and identification of *fln* seedlings was not possible (Figure 3B, top). After 2 days, wild-type cotyledons were reflexed, fully green and almost completely expanded, and the pale *fln* cotyledons were evident (Figure 3B, middle). These results indicate that FLN1-HA expression must occur very early in seedling development in order to rescue the mutant phenotype.

### Loss of *fln1* or *fln2* specifically disrupts PEP-dependent transcription

**Figure 4:**
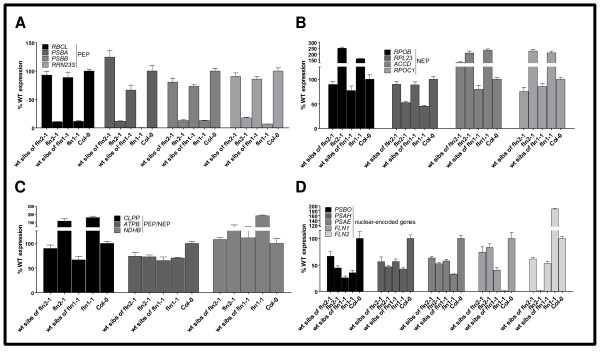
** Expression of chloroplast encoded genes in*fln*seedlings.** qRT-PCR analysis of the indicated gene transcript levels was performed in 7-day-old seedlings grown as in Figure 1B. Four classes of genes were examined, PEP-dependent **(A)**, NEP-dependent **(B)** PEP- and NEP-transcribed **(C)**, and nuclear-encoded **(D)**. Data shown are the mean ± standard error of the mean (SE) of a representative experiment (n = 3). Expression levels are presented as the percentage relative to wild type.

To explore the role of each FLN in chloroplast transcription, chloroplast-encoded mRNA levels were determined in *fln1-1* and *fln2-1*. Previously, the effect of reduced *FLN* expression by inducible RNA-interference was investigated in *Arabidopsis* and tobacco leaves [15], but had not been determined in stable *fln* null plants. Here, we examined levels of mRNA in our *fln* null alleles for the three classes of chloroplast-encoded genes: PEP-dependent (Class I, Figure 4A), NEP-dependent (Class III, Figure 4B), both PEP- and NEP- transcribed C(Class II, Figure 4) and for nuclear genes encoding plastid-localized proteins (Figure 4D). RNA from 7-day old *fln* seedlings and their phenotypic wild-type siblings (a combination of *FLN* and *FLN/fln* genotypes) were subjected to quantitative real-time RT-PCR (qRT-PCR) analysis (Figure 4).

Expression of PEP-dependent genes *rbcL*, *psbA*, *psbB*, and *rrn23S* was strongly reduced in both *fln1-1* and *fln2-1* seedlings compared to Col-0 and to their respective phenotypically wild-type siblings (Figure 4A). Transcripts for NEP-dependent genes *rpoB*, *accD*, and *rpoC1*, on the other hand, were slightly elevated in *fln* seedlings, except for *rpl23* transcripts with only 50% of wild type expression in the mutants. *clpP*, *atpB*, and *ndhB* are transcribed by both PEP and NEP. Both *clpP* and *ndhB* mRNAs were slightly up-regulated in the mutants, while *atpB* mRNA was similar in both *fln* mutants to their respective wild type siblings (Figure 4C).

Expectedly, mRNAs for *FLN1* and *FLN2* were absent in cDNA prepared from their respective mutants. Interestingly, *FLN2* mRNA is two-fold higher in *fln1-1* than Col-0, but the opposite is not true, *FLN1* mRNA is not elevated in *fln2-1* (Figure 4D). For other nuclear-encoded chloroplast-localized proteins, *fln* and wild-type siblings had similar transcript levels, approximately a 50% reduction compared to Col-0 (Figure 4D). This reduction likely results from the mixed genotypes of the phenotypically wild type sibling seedlings consisting of *FLN/FLN* and *FLN/fln1-1* genotypes. In summary, only PEP-dependent transcripts were consistently and severely down-regulated in *fln* seedlings, indicating that FLNs are positive regulators of TAC complex function.

### FLN proteins are not required for plant cell elongation in the dark

**Figure 5:**
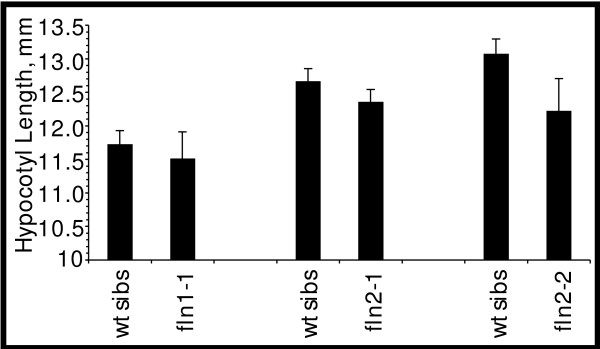
** Hypocotyl length of 7 day old dark-grown*fln*mutants.** Progeny from *FLN/fln* parents were grown in the dark for 7 days at 20^o^ C. Measurements of *fln* hypocotyls are shown next to their respective phenotypically wild-type siblings and combined for two different growth experiments. No *fln* mutant was significantly different from wild type siblings using a Student’s *T*-test (α=0.05). Bars are se. (35≥ n ≥27).

Given the strong dependence on *FLN* expression for wild-type growth of light-grown seedlings, we sought to ascertain if FLN proteins are required for skotomorphogenesis. We examined hypocotyl elongation in dark-grown 7 day-old seedlings from *FLN/fln* parents. All seedlings had elongated hypocotyls, an apical hook and unexpanded cotyledons (data not shown). We measured hypocotyl length of etiolated seedlings to assess if cell expansion in the dark was affected (Figure 5). *fln* hypocotyl lengths were not significantly shorter than those of their corresponding wild-type siblings as assessed by a Student’s *t*-test (p-values for comparison to wild-type siblings: *fln1-1*, 0.607; *fln2-1*, 0.202; and *fln2-2*, 0.0821; α = 0.05).

Dark-grown seedlings were then exposed to light. Cotyledons of *fln1-1* mutants never greened, but did unhook in the light. *fln2* seedlings unhooked and turned pale green, eventually resembling cotyledons of *fln2* mutants grown under continuous light. Thus, prior expansion in the dark did not change the phenotype of *fln* cotyledons in the light. We therefore conclude that neither FLN1 nor FLN2 are required for skotomorphogenesis.

### FLN proteins are required for normal plastid development

**Figure 6:**
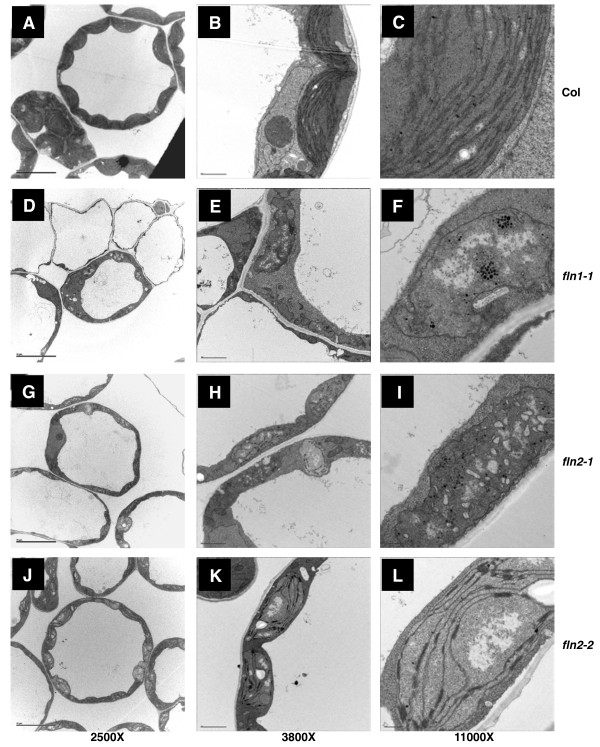
** Chloroplast ultrastructure in*fln*mutants.** Cotyledons from 7-day old seedlings were fixed and sectioned for TEM imaging. **(A)** to **(C)** Col-0, **(D)** to **(F)***fln1-1*, **(G)** to **(I)***fln2-1*, **(J)** to **(L)***fln2-2*. Scale bars for first, second, and third columns are 10, 2, and 0.5 μm, respectively.

To investigate plastid development in *fln1* and *fln2* seedlings, we examined chloroplast ultrastructure in cotyledons of 7 day-old light-grown seedlings grown on GM by transmission electron microscopy (TEM) (Figure 6). Columbia (Col-0) cells contained a population of morphologically homogenous chloroplasts (Figure 6A). Wild-type chloroplasts are typified by stacks of thylakoid grana membrane connected via stromal thylakoid membranes with a few starch granules and plastoglobuli interspersed throughout the stroma (Figure 6C). Consistent with *fln1* mutants having a more severe morphological phenotype (Figure 1), cells of *fln1-1* were extremely vacuolated and contained chloroplasts with more extreme defects (Figure 6D, E) than those of *fln2* cells (Figure 6G, J). Almost all * fln1* chloroplasts lack internal membrane structures with a large number of densely staining plastoglobuli. (Figure 6F).

In contrast, *fln2* cells contained a population of chloroplasts with a range of defects (Figure 6G, J). In both *fln2* alleles, chloroplasts have highly disrupted internal membrane structures compared to wild-type Col-0 (Figure 6H, K). The majority of *fln2-1* and *fln2-2* chloroplasts were missing thylakoid membranes, highly vacuolated, containing many more plastoglobuli than wild-type, and filled with unidentifiable suborganellar structures (Figure 6I). Other chloroplasts contained some stromal thylakoid membrane and a few grana stacks (Figure 6L). In summary, *fln2-1* chloroplasts were either lacking any typical membrane structures or contained only a few thylakoid membranes and grana stacks. *fln2-2* chloroplasts, on the other hand, had some contiguous stromal thylakoid membrane forming a reticulum with reduced granal stacks (Figure 6L).

**Figure 7:**
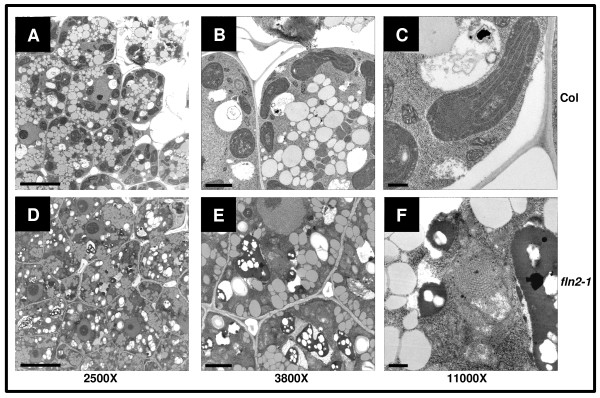
** Etioplast ultrastructure in*fln2-1*mutants.** Cotyledons from 3-day old dark grown seedlings grown at 20° C were fixed and sectioned for TEM imaging. **(A)** to **(C)** Col-0, **(D)** to **(F)***fln2-1*. Scale bars for first, second, and third columns are 10, 2, and 0.5 μm, respectively.

While mature chloroplasts of *fln* mutants fail to develop properly and mutants have severe growth defects in the light, cell elongation in the dark is largely unaffected (Figure 5). These observations lead to us investigate whether etioplast development was affected in *fln* mutants. Only *fln2* could be analyzed because *fln1* homozygous seed cannot be obtained [*fln1-1* is seedling lethal (Figure 1)], and *fln1-1* segregants cannot be phenotypically distinguished from *FLN1* and *FLN1/fln1-1* siblings in the dark (Figure 5). *fln 2–1* etioplasts were examined from cotyledons of 3-day old dark-grown seedlings by TEM (Figure 7). For both Col-0 and *fln2-1* dark-grown cells, the cytoplasm was dense with multiple small vacuoles and oil bodies (Figure 7A, D). *fln2-1* cells also contained subcellular components that did not fix during the preparation of the samples, leaving holes in the sections (Figure 7E). Etioplasts in Col-0 could easily be identified and stained densely (Figure 7B), but *fln2-1* etioplasts stained less densely and were difficult to discern from other cellular contents (Figure 7E). Col-0 etioplasts contained obvious prolamellar bodies with stromal strands of thylakoid membrane extending from them, as classically observed (Figure 7C). *fln2-1* etioplasts did contain prolamellar bodies, but were atypical and lacked any obvious associated stromal thylakoids (Figure 7f). Altogether, these data indicate that *FLN2* is not only required for chloroplast development in the light, but also for development of etioplasts in the dark.

### FLNs function additively in plant growth and development

**Figure 8:**
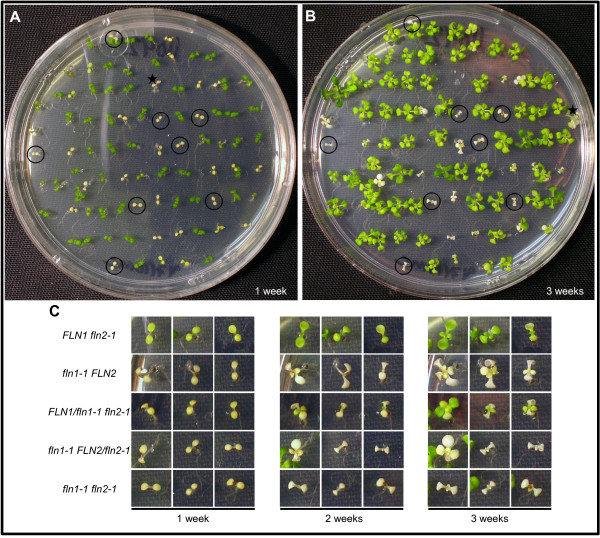
*** fln1 fln2*double mutant analysis.** F_2_ progeny from *FLN1/fln1-1 FLN2/fln2-1* parents are shown. **(A)** and **(B)** F_2_ seeds were plated on GM and grown for three weeks with images taken at 1 week (A), 2 weeks, and 3 weeks (B). Pale and white seedlings were sacrificed for genotyping. Individuals identified as *fln1 fln2* are circled. One individual accidentally moved on the plate during the growth period and is denoted with a star. **(C)** Left, middle and right sets of pictures, magnification of seedlings from the plate shown in (A), after 2 weeks growth, and from the plate in (B), respectively. Three representative seedlings are shown for each indicated genotype, with their phenotypes shown at different ages.

To determine the effect on seedling growth when both FLN proteins are absent, we crossed *fln2-1* homozygotes to *FLN1/fln1-1* heterozygotes to obtain *fln1 fln2* double homozygous mutants. Seeds from *FLN1/fln1-1 FLN2/fln2-1* plants were plated and growth monitored over a three-week period. One week-old pale/chlorotic seedlings were marked and photographed (Figure 8A, B). The same plate was incubated for two additional weeks, after which these plants were sacrificed for *fln1-1* and *fln2-*1 genotyping. Double *fln1 fln2* mutants were identified (circled, Figure 8A, B), and recovered with the expected 1/16 frequency in a large F_2_ population (χ^2^ = 1.142, p-value = 0.2853, α = 0.05).

Also among the one week-old pale seedlings were single mutant siblings and siblings heterozygous at one locus and homozygous at the other (Figure 8C). Three different seedlings of each genotype, representative of the phenotypes observed, are shown at different ages. Single *fln2-1* seedlings have pale cotyledons, and a few green true leaves by three weeks of growth; unlike on soil, other leaves remain pale (Figure 8C, first row). As observed on soil (Figure 1), single mutant *fln1-1* cotyledons are white and totally void of visible chlorophyll. Unlike on soil, *fln1-1* seedlings grown on media supplemented with 1% sucrose produce true leaves. These leaves are white and never green (Figure 8C, second row).

Analysis of *FLN1/fln1-1 fln2-1* and *fln1-1 FLN2/fln2-2* plants revealed haplo-insufficiency for FLN function in these genotypes. *FLN1/fln1-1 fln2-1* seedlings are more similar in appearance to the more severe single *fln1-1* rather than to single *fln2-1* after one week of growth, and the cotyledons never green. True leaves for this genotypic class emerge pale green, but are less expanded than those of *fln2-1*. By three weeks, some *FLN1/fln1-1 fln2-1* plants resemble *fln2-1*, while the majority has a more severe phenotype (Figure 8C, third row). *fln1-1 FLN2/fln2-1* seedlings appear like *fln1-1* after seven days of growth, but by 14 days a fraction of this genotypic class begins to have shriveled cotyledons, while others continued to look like *fln1-1* (Figure 8C, fourth row). Even after three weeks, the more severe individuals never expand their true leaves, and resemble the *fln1-1 fln2-1* double mutant (Figure 8C, bottom row).

Double mutant *fln1-1 fln2-1* plants are indistinguishable from their *fln1-1* siblings after 7 days of growth, but are clearly different after three weeks (Figure 8c, fifth row). All double mutants produce one set of very small, white true leaves visible by two weeks, but these never expand. The double mutant and a subset of *fln1-1 FLN2/fln2-1* seedlings cannot be distinguished from each other phenotypically, and together demonstrate that seedlings lacking all or with insufficient FLN activity are seedling lethal even when supplemented with sucrose. This result suggests that FLN function, in some capacity, is required for development even when sucrose is present. This genetic analysis also suggests that FLN1 and FLN2 probably do not have functionally specificity, but rather work additively to regulate gene expression.

## Discussion

Two proteins with predicted domains related to phosphofructokinases in the pfkB family are found in plastid TAC complexes from multiple plant species, but their roles remain enigmatic. *E. coli* possesses two phosphofructokinases, and *PfkB/Pfk-2* was originally discovered as a suppressor of a *PfkA/Pfk-1* mutation [28,29]. While both PfkA and PfkB are catalytically active, they are not similar in sequence and have different allosteric regulation [30-32]. Thus, kinases with sequence similarity to PfkB became known as pfkB-type kinases. Other PfkB-type kinases phosphorylate a variety of substrates including fructose, fructose-6-phosphate, ribose, adenosine, phosphomethylpyrimidine, and tagatose-6-phosphate [33], but many remain poorly characterized. To unravel the roles of pfkB-type kinases in plant growth and development we have taken a reverse genetics approach to assess the phenotypes of pfkB-type kinase loss-of-function mutants, beginning with genes encoding two closely-related FRUCTOKINASE-LIKE PROTEINs, FLN1 and FLN2, identified as components of the PEP TAC complex [9,12,14]. Using semi-quantitative and qRT-PCR, we demonstrate that these *fln* insertion alleles do not produce detectable mRNA and are null alleles, and through complementation and characterization of multiple alleles, indicate that the observed phenotypes result from loss of *fln* expression. These null alleles were used to separately assess FLN’s individual and collective contribution to plant growth and development.

The work initially describing FLNs, addressed their roles using RNAi in adult plants [14,15]. Silencing of either *FLN1* or *FLN2* caused similar chlorosis in young and developing *Arabidopsis* rosette leaves [15]. In contrast, our *FLN* loss-of-function analysis revealed unequal roles for FLN1 and FLN2. Accordingly, we observed that chloroplasts of *fln1* plants are more severely affected than those of *fln2*. The differences between the two experiments might be because the differential contributions of FLN1 and FLN2 are greater during early seedling growth than in rosette leaves. The effect of reduced *FLN* expression was also assessed previously in tobacco. RNAi of only *FLN1* in tobacco resulted in chlorosis, consistent with our proposed greater role for FLN1. Chloroplast ultrastructure was not examined in the *Arabidopsis* RNAi lines; however, the authors did examine chloroplasts of *FLN1* RNAi in tobacco cells [15]. Chloroplasts in *FLN1* RNAi tobacco were very similar to those in *Arabidopsis fln1* plants reported here, indicating that loss of this protein in both tobacco and *Arabidopsis* results in similar plastid defects.

FLN’s localization to TAC complexes suggests that PEP loss of function mutants should have similar phenotypes. The PEP holoenzyme is similar in composition to eubacterial RNA polymerases and comprised of four different subunits α_2_ββ′β′′ encoded by the plastid genes *rpoArpoBrpoC1*, and *rpoC2*, respectively. PEP-dependent transcription additionally requires nuclear-encoded sigma factors to ensure the correct transcription start site [reviewed in [34]]. Mutations in *rpo* genes in tobacco result in albinism and severe defects in plastid development [6,35]. *rpo*^*-*^ plants can grow only when supplemented with sucrose; however, these plants resemble wild-type plants morphologically with the exception of pigmentation loss, and can develop normally, flower and set seed [35]. Here we show that *fln1* plants also require sucrose for growth, but are not identical to *rpo*^*-*^ plants. *fln1* grows and flowers on sucrose, but plants are extremely small and infertile (data not shown). *fln2* plants, on the other hand, remain autotrophic with more normal chloroplasts than found in either *fln1* or *rpo*^*-*^ both of which are lacking thylakoid membranes and are highly vacuolated [6,35]. The *fln1-1 fln2-1* double mutant is seedling lethal even when grown on sucrose and only one set of two true leaves emerges without subsequent expansion before dying. This result, in light of the viability of *rpo*^*-*^ plants grown on sucrose, suggests that the FLNs may serve another function in addition to their role in PEP complexes or that loss of FLNs is more toxic than loss of core PEP subunits. A challenge to this interpretation is that no *rpo*^*-*^*Arabidopsis* have been described. Those plants may be more severe than the tobacco mutants and more closely resemble the *fln* double mutant. A FLN substrate remains elusive [15], therefore, a full understanding of their role both inside and out of the TAC complex awaits the identification of a substrate. One approach to dissect a function independent of the TAC complex would be to assess phenotypes and chloroplast-encoded transcript levels in *fln* nulls expressing FLNs that do not localize to TAC complexes. However, such mutations have not been identified to date.

In this work, we also sought to assess the role of FLN proteins during skotomorphogenesis. We evaluated etioplast structure in *fln2* dark-grown seedlings. We were unfortunately unable to examine etioplasts in *fln1* seedlings because seedling lethality prevented collection of a population of homozygous seed and *fln1-1* seedlings are indistinguishable from wild-type siblings when grown in the dark. However, based on the mild defect in *fln2* etioplasts and the normal skotomorphogenesis of both *fln1* and *fln2* plants, we conclude that FLNs have a more prominent role in plastid development in the light than mediating growth in the dark.

FLNs appear to be regulated by the thioredoxin system that modulates enzyme activities in response to illumination, which for FLNs, requires a specific thioredoxin, TRX-z [15]. *trx-z* null plants are viable and develop morphologically normal leaves abeit without chlorophyll when supplied with sucrose [15]. In contrast, complete loss of FLN expression in a *fln1 fln2* background results in a seedling lethal phenotype, even when supplied with exogenous sucrose. The differences in the growth abilities between *trx-z* and *fln1 fln2* mutants on sucrose synthetic media suggests that there is some FLN activity in *trx-z* plants and hence, redox regulation by TRX-z is not strictly required for FLN activity.

Consistent with their location in TAC complexes [15] and previous RNA analyses with RNAi plants [15], single *fln* mutants dramatically affect PEP-dependent chloroplast RNA accumulation, while most of the NEP and NEP/PEP-dependent mRNAs slightly accumulate or are not affected. These findings are in line with previous observations that disruption of PEP-dependent transcription results in an elevation of class III and some class II chloroplast genes (reviewed in [36]). One exception in our experiments was *rpl23*, a NEP-dependent gene whose mRNA was reduced in *fln* seedlings. A recent study showed that *rpl23* transcript level is reduced when PEP is inhibited during germination [37], suggesting that this gene can be transcribed by both NEP and PEP during this phase of seedling establishment. Arsova *et. al* 2010 only examined NEP-dependent transcripts in mature rosette leaves, and did not include *rpl23*. This study adds new insight into the requirement of FLNs for PEP activity during early seedling growth.

PEP-dependent transcription is maximal during greening, although a role early in germination is proposed [37,38]. A recent report suggests that PEP is present and active during imbibition; however, PEP transcribes only genes encoding ribosomal proteins during this stage of development as revealed by using tagetin, a PEP-specific inhibitor. PEP begins to transcribe photosynthesis related genes within 24 hours of imbibition, prior to completion of germination [37]. The ability of FLN1-HA to complement the *fln1* phenotype when expressed after seed stratification and after one day of germination suggests that FLN activity is not required early, but rather as the seedlings are developing photosynthetic competency. These plants do, however, express FLN2, so it is possible that FLN2 provides sufficient activity early, but is insufficient during rapid chloroplast maturation. The plastids in *fln1* cotyledons do not resemble chloroplasts (Figure 6), and are missing most internal membranes. Addition of FLN1-HA to these plastids by DEX-induction cannot rescue the pale phenotype, implying that the effects of reduced FLN are not reversible. In other words, once photomorphogenesis has proceeded past a specific point, addition of FLN1 cannot restore normal greening. These observations are consistent with the inability of grossly mis-developed/damaged chloroplasts to reorganize and repair themselves, after development from either etioplasts or pro-plastids.

PEP core subunits are found in soluble PEP complexes and in the insoluble TAC complexes associated with plastid DNA [9]. TACs are estimated to contain 40–60 different proteins in addition to PEP subunits [9]. Although mutant analysis has not been reported for all components of TAC, plants with mutations in PEP and pTAC encoding genes have diverse pleiotropic phenotypes, but all share, to some degree, loss of pigmentation and abnormal plastid development. To our knowledge, the following TAC components have been assessed by loss of function analysis: pTAC1/WHIRLY1 [39], pTAC2 [9], pTAC4/VESICLE-INDUCING PROTEIN IN PLASTIDS [40], pTAC6 [9], pTAC11/WHIRLY3 [39], pTAC12/HEMERA [9,41], IRON SUPEROXIDE DISMUTASE 1 and 3 [42], DNA GYRASE [43,44], THIOREDOXIN Z [14,15], URIDINE DIPHOSPHATE-*N*-ACETYLMERAMIC ACID LIGASE E [45], pTAC14 [46], GENOMES UNCOUPLED 1 [47,48], PLASTID REDOX INSENSITIVE 2 [49] and FRUCTOKINASE-LIKE PROTEIN 1 and 2 [[11,15] and this work]. With the exception of WHIRLY, DNA GYRASE, GENOMES UNCOUPLED 1, and PLASTID REDOX INSENSITIVE 2, all mutants listed above require an exogenous carbon source for viability. WHIRLY mutants have a low penetrance of chlorotic variegation, and these mutants are fully autotrophic. DNA GYRASE mutants, similar to *fln* double mutants, only produce two true leaves that fail to expand, with no further growth even when supplemented with sucrose.

While the exact mechanism of FLN action remains to be elucidated, our genetic analysis points to an overlapping role of FLN1 and FLN2 in terms of plastid development and plastid-specific gene expression. Phylogenetic analysis of plant pfkB-type kinases revealed that the FLNs clade with enzymes shown to have fructokinase activity and are more distantly related to known adenosine kinases [15]. Attempts to identify a FLN substrate were unsuccessful using recombinant FLN and a variety of sugars [15]. FLNs could serve both an enzymatic function not yet identified and/or additionally act as a metabolic sensor in PEP complexes to coordinate gene expression with metabolic status of the plastid. Proteins with dual function in metabolism and signaling have been characterized across all kingdoms of life [reviewed in [50]], and *Arabidopsis* HEXOKINASE1 has been shown to chromatin immunoprecipitate with nuclear DNA and other proteins required for glucose signaling [51].

## Conclusions

Our analysis of *fln* loss-of-function mutants further substantiates the RNAi phenotypes reported previously for *Arabidopsis* and tobacco, and reveals an essential role for these proteins in plant growth and development. Transcripts of PEP-dependent chloroplast genes are severely reduced in *fln* seedlings by qRT-PCR analysis, indicating that FLN proteins are indispensible components of the TAC complex. Genetic analyses show that *FLN1* plays a more dominant role than *FLN2*; single mutant analysis showed FLN1 to be required for autotrophic growth; double mutant analysis revealed that complete loss of FLN function results in seedling lethality even on an exogenous carbon source. This analysis demonstrates that seedlings are extremely sensitive to FLN levels.

## Methods

### Plant materials and growth conditions

All plants are *Arabidopsis thaliana*, ecotype Columbia-0 (Col-0) unless otherwise noted. At3g54090 T-DNA insertion line GK-443A08 (*fln1-1*) was obtained from The European *Arabidopsis* Stock Centre (http://Arabidopsis.info/) [52]. T-DNA insertion lines for At1g69200 were SALK_008812 (*fln2-1*) obtained from the *Arabidopsis* Biological Resource Center (ABRC) (http://abrc.osu.edu/) [53] and FLAG_110F06 (*fln2-2*, in Wassilevskija (WS-4) ecotype) obtained from INRA *Arabidopsis thaliana* Resource Centre for Genomics (http://www-ijpb.versailles.inra.fr/en/sgap/equipes/ variabilite/crg/index.htm) [54]. A putative third allele from ABRC [SALK_005734 (*fln2-3*)], was found to have the identical insertion site as SALK_008812 (Additional file 1: Figure S1) and is likely an inadvertent duplicate of the same insertion event. It exhibited the same phenotypes as *fln2-2*. PCR genotyping primers are listed in Additional file 2: Table S1. All plants lines were F_2_ progeny from a backcross (BC_1_) to Col-0. Growth media (GM) for plants grown in plates was prepared as previously described [55]. Seeds were surface sterilized in 30% bleach, 0.1% Triton-X 100 prior to plating. All plate-grown seedlings were grown at 20^o^ C under constant white light at 40–50 μmol/sec^-1^m^-2^.

For hypocotyl measurements, progeny from *FLN/fln* were grown on solid GM in the dark for 7 days. Seedlings were then transferred to new plates, imaged, their positions noted, and hypocotyl lengths determined from this image. To identify which hypocotyls were *fln*, seedlings were placed under lights and allowed to grow an additional 7 days. Homozygous *fln* hypocotyls were identified based on white/pale phenotype of cotyledons and true leaves. Measurements were obtained using Image J 1.36 (http://rsb.info.nih.gov/ij/).

### RT-PCR analysis of *FLN* transcripts in insertion lines

BC_1_ F_2_ seeds segregating for each *fln* allele were plated on GM and grown for 7 days. Homozygous mutants were identified by phenotype, harvested, and stored in liquid N_2_ for RNA extraction. RNA extraction was performed using the RNeasy plant mini kit (Qiagen). DNase digestion was carried out on the column prior to RNA elution to eliminate contaminating genomic DNA. For *fln1* and *fln2* experiments, 2.5 and 2.8 μg total RNA, respectively, was used in reverse-transcriptase (RT) reactions using SuperScript III RT (Invitrogen, Carlsbad, CA) and oligo(dT) primer to synthesize cDNA. cDNA was then used as a template to amplify *FLN* full-length coding sequences (CDS). PCR products were 1437, 1851, and 1446 bp for *FLN1*, *FLN2*, and *UBIQUITIN10* (*UBQ10*) respectively. *UBQ10* was used a control to ensure that equal RNA was used for all cDNA synthesis reactions. The *FLN1* product included the entire 5’UTR as annotated and ended at the stop codon sequence, while the *FLN2* was the coding sequence from start to stop codon sequence. Primers used for RT-PCR experiments are listed in Additional file 2: Table S1.

### qRT-PCR analysis of chloroplast gene expression

Plants were grown and RNA extracted as described above for RT-PCR. cDNA was prepared from 2 μg total RNA using the Maxima first strand cDNA synthesis kit (Fermentas). 5 μL of a 1:400 cDNA dilution was used as template for qRT-PCR. Real time PCR was performed using a CFX384^TM^ Real-Time System (BIO-RAD) using SYBR Green (Invitrogen). The following standard thermal profile was used for all PCR reactions: 95 °C for 3 min, 40 cycles of 95 °C for 10s and 60 °C for 1 min. Primers for *psbOpsaHpsaE*, and *ndhB* were as previously published [15]. Additional primers are listed in Additional file 2: Table S1. Expression levels were normalized to 18S rRNA content.

### Transmission electron microscopy

Seedlings were grown on GM plates as described above. Light grown seedlings were 7 days old, and dark grown seedlings were 3 days old. Cotyledons were fixed in Karnovsky’s fixative [56] using a microwave [57], rinsed in 0.1 M PBS and post-fixed in buffered 1% osmium Tetroxide for 2 hours. Samples were then incubated in 0.1% tannic acid (aqueous) for 30 minutes before dehydrating [58-60] in ascending concentrations of ethyl alcohol (ETOH) for 20 minutes each step (30%-50%). They were then stained with 2% uranyl acetate in 50% ETOH [61]. Dehydration was completed with 70%, three changes of 95% and two changes of 100% ethanol for 20 minutes each [57]. Pure epoxy resin (Epon/Araldite) replaced the final ethanol step and was infiltrated overnight [62]. The samples were then carefully placed in the bottom of a flat embedding mold (Electron Microscopy Sciences, PA, USA), and the capsules filled with fresh resin and polymerized overnight in a 70^o^ C oven. Sections were cut on a Leica Ultracut UCT Ultramicrotome using a diamond knife (Diatome, Switzerland, EMS U.S.A. distributor). The samples were viewed and images were taken using a Philips CM120 Biotwin TEM, (FEI Company, Hillsboro, OR, USA) using a Gatan MegaScan 794/20 digital camera (2 K X 2 K) (Pleasanton, CA, USA). All work was completed in the Electron Microscopy Laboratory, Department of Medical Pathology and Laboratory Medicine, School of Medicine, University of California at Davis.

### Genetic Complementation of *fln1-1* mutants and inducible expression of FLN1

We obtained a full-length clone of the *FLN1* (At3g54090.1) coding sequence (Clone U17866) from the ABRC [63] in a pENTR/SD/D-TOPO backbone. To clone this ORF without a stop codon for making C-terminal tagged translational fusions, we used U17866 as a template using Phusion high-fidelity DNA polymerase (NEB). Primers used are listed in Additional file 2: Table S1. The PCR product was gel-purified and recombined into pDonr201 using a GATEWAY Technology BP-clonase reaction (Invitrogen). The ORF was sequenced verified then recombined into pBAV154 plant transformation vector [27] according to manufacturer’s directions. The final plant expression cassette after recombination allows for dexamethasone (DEX)-induced expression of FLN1-HA. The plant transformation vector was introduced into *Agrobacterium tumefaciens*, strain AGL1, which was then used to transform *fln1-1* heterozygotes using the floral dip method [64]. Transformants were selected by spraying progeny with a 0.578% solution of Finale (1% glufosinate-ammonium, Bayer CropScience http://www.bayercropscience.com/); resistant T_1_ plants were genotyped for the *fln1-1* insertion and 14 heterozygotes (independent transformation events) carried forward to the T_2_ generation. Because only *fln1-1* heterozygotes survive on GM supplemented with sulfadiazine, T_2_ seeds from these 14 lines were plated on 5.25 μg/mL sulfadiazine sodium (Sigma-Aldrich, S6387) to select for *fln1-1* heterozygotes. Resistant individuals were transferred to soil and sprayed with Finale to determine the number of *FLN1-HA* transgenes present in each line. Only lines segregating 3:1 for glufosinate resistance were carried forward. T_3_ seeds from these T_2_ families were sown on soil and again selected for glufosinate resistance to determine which T_2_ family individuals were fixed for the *FLN1-HA* transgene. T_3_ seeds homozygous for the *FLN1-HA* transgene and segregating for *fln1-1* were used dexamethasone-dependent complementation experiments. Seed were plated on varying concentrations of dexamethasone and the number of seedlings with green or pale cotyledons were scored after 7 days. For the developmental time course, seed were plated, stratified for 2 days at 4 °C, then placed at room temperature (RT). Dexamethasone was added to a plate the same day or after 1 or 2 days at RT and scored for green cotyledons after 7 days.

### Western blot analysis

Total protein extracts were prepared from seedlings grown for complementation experiments described above. Tissue was homogenized in a 1:1 seedling weight (mg) to buffer (μl) ratio using 5x Laemmli sample buffer (125 mM Tris pH 6.8, 20% SDS, 10% β-mercaptoethanol, 20% glycerol, 0.2 mg/mL bromophenyl blue). After homogenization, lysates were boiled 5 min and cleared by centrifugation at 16,000xg for 20 min. Proteins were separated by SDS-PAGE on 8% gels, and transferred to PDVF Immobilon-P membrane (Millipore, Billerica, MA, USA) for immunoblotting. Membranes were blocked with BLOTTO [5% non-fat dry milk in TBS-T (50 mM Tris, 200 mM NaCl, 0.1% Tween-20), 0.165% antifoam Y emulsion (Sigma)] for 15 min, then immunoblotted with 1:1000 anti-HA-peroxidase (3 F10 rat monoclonal antibody, Roche Diagnostics, Mannheim, Germany) in BLOTTO for 2 hr. Membranes were washed in TBS-T, then developed with Amersham ECL Plus western detection system (GE Healthcare, Buckinghamshire, United Kingdom) and X-ray film following the manufacturer’s instructions.

## Abbreviations

NEP, Nuclear-encoded polymerase; PEP, Plastid-encoded polymerase; FLN1 and FLN2, FRUCTOKINASE-LIKE PROTEIN; TAC, Transcriptionally active chromosome; qRT-PCR, Quantitative real-time RT-PCR; TEM, Transmission electron microscopy; ADK, Adenosine kinase.

## Competing interests

The authors declare they have no competing interests.

## Authors’ contributions

JG and JCallis conceived the experiments, and JG, JMPR and JCallis performed the experiments. JChory participated in the design of the qRTPCR study. JG wrote the first draft, which was edited by JCallis and JChory. All authors read and approved the final manuscript.

## Supplementary Material

Additional file 1: Figure S1 Sequence of T-DNA borders in *fln* alleles. The T-DNA specific bands from PCRs were sequenced from the left border of the T-DNA for the three different lines. Orientation of the T-DNA is denoted by “L” for left and “R” for right borders. Boxed sequence is additional DNA not in T-DNA or at locus prior to insertion. Right border sequence at insertion site was not determined. Because *fln2-1* and *fln2-3* have two inverted T-DNA insertions in tandem, sequence on each side of the insertion could be determined. Unboxed sequence is present in endogenous locus. Click here for file

Additional file 2: Table S1 DNA Primer sequences used for PCR. Contains a list of primer sequences, by purpose, used in this study. Click here for file
