# Supplementary material for: The plastid-localized pfkB-type carbohydrate kinases FRUCTOKINASE-LIKE 1 and 2 are essential for growth and development of Arabidopsis thaliana
Source: BMC Plant Biol. 2012 Jul 8;12:102. doi: 10.1186/1471-2229-12-102 (PMC3409070; doi:10.1186/1471-2229-12-102)
Supplement: Additional file 1: Figure S1 — Sequence of T-DNA borders in fln alleles. The T-DNA specific bands from PCRs were sequenced from the left border of the T-DNA for the three different lines. Orientation of the T-DNA is denoted by “L” for left and “R” for right borders. Boxed sequence is additional DNA not in T-DNA or at locus prior to insertion. Right border sequence at insertion site was not determined. Because fln2-1 and fln2-3 have two inverted T-DNA insertions in tandem, sequence on each side of the insertion could be determined. Unboxed sequence is present in endogenous locus. [file 1471-2229-12-102-S1.pdf]

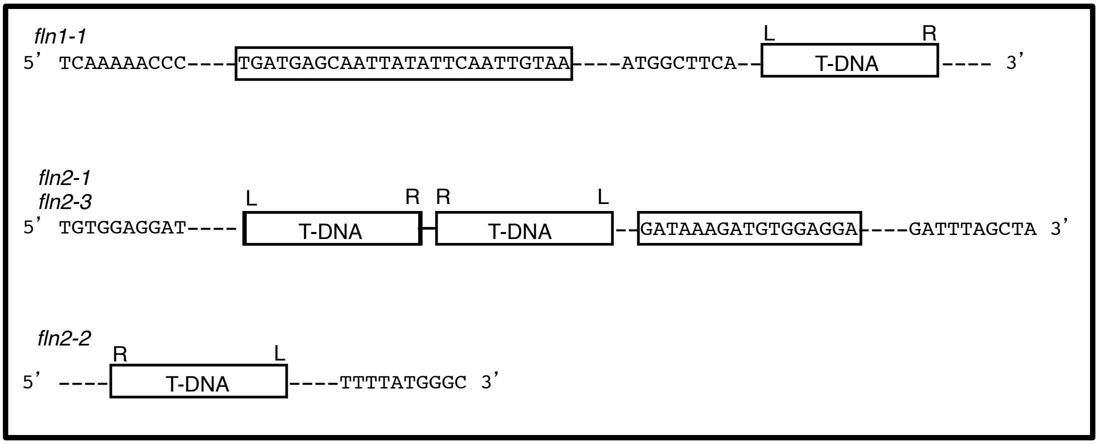
fln1-1
5' TCAAAAACCC----TGATGAGCAATTATATTCAATTGTAA----ATGGCTTCA--
L
T-DNA
R
---- 3'
fln2-1
fln2-3
5' TGTGGAGGAT----
L
T-DNA
R
R
T-DNA
L
--GATAAAGATGTGGAGGA----GATTTAGCTA 3'
fln2-2
5' ----
R
T-DNA
L
----TTTTATGGGC 3'
