# Supplementary material for: The plastid-localized pfkB-type carbohydrate kinases FRUCTOKINASE-LIKE 1 and 2 are essential for growth and development of Arabidopsis thaliana
Source: BMC Plant Biol. 2012 Jul 8;12:102. doi: 10.1186/1471-2229-12-102 (PMC3409070; doi:10.1186/1471-2229-12-102)
Supplement: Additional file 2: Table S1 — DNA Primer sequences used for PCR. Contains a list of primer sequences, by purpose, used in this study. [file 1471-2229-12-102-S2.doc]

| **Purpose** | **Primer Sequence, 5’ to 3’** |
| --- | --- |
| **Genotyping** | |
| Salk T-DNA left border | TGGTTCACGTAGTGGGCCATCG |
| FLAG T-DNA left border | CTACAAATTGCCTTTTCTTATCGAC |
| GABI-Kat T-DNA left border | ATATTGACCATCATACTCATTGC |
| *fln1-1* gene specific left | CAAATAGCGAGTCCTCAGGTG |
| *fln1-1* gene specific right | GATCAATTCCCACAAGGAAGC |
| *fln2-1* gene specific left | AGAGGTGAAAGATGGAATGGG |
| *fln2-1* gene specific right | TCAATGTTCCAAATCTCCAGG |
| *fln2-2* gene specific left | CAACGAAGAAGAAGGTTGTGG |
| *fln2-2* gene specific right | ATGACGTCTGCAAGATTCCAC |
| *fln2-3* gene specific left | GTAGAACATTTTCGCCTGCAC |
| *fln2-3* gene specific right | GGTTGTGGGAAAGAAACCATC |
| **RT-PCR** | |
| *FLN1* left | agtggtgtcaaaaaccc |
| *FLN1* right | CTACCACATTGATGGAACAT |
| *FLN2* left | ATGGCGTCTCTCTCCTTCACCC |
| *FLN2* right | TCATAAACTACCATCTTCAAAC |
| *UBQ10* left | TCAATTCTCTCTACCGTGATCAAG |
| *UBQ10* right | TTACATGAAACGAAACATTGAACTT |
| Gateway Cloning of FLN coding sequences | |
| *FLN1* left | GGGGACAAGTTTGTACAAAAAAGCAGGCTTGATGGCTTCACTTCTTATTTTC |
| *FLN1* right | GGGGACCACTTTGTACAAGAAAGCTGGGTCCCACATTGATGGAACATAAAC |
| **qRT-PCR** | |
| *FLN1* left | ACTTGTCCTGAGATGTTTGAG |
| *FLN1* right | CTAACTGCACCAATTGTCCAC |
| *FLN2* left | CCTCAAAGCCTTATGATGAACC |
| *FLN2* right | CTTGTACTCCCTTTCCTCTACTG |
| *rbcL* left | CCTCAAAGCCTTATGATGAACC |
| *rbcL* right | TGAGTTTCTTCTCCTGGAACGG |
| *psbA* left | CGTCTTTACATTGGATGGTTTGG |
| *psbA* right | CAGAAGTTGCGGTCAATAAGG |
| *psbB* left | TCGTGCGACTTTGAAATCTGA |
| *psbB* right | CAACCTCTTGGGCTGCTACG |
| *rrn23S* left | TTATGCTCTGACCCGAGTAGCA |
| *rrn23S* right | CTGATTCACACGGGATTCCAC |
| *clpP* left | TGGGTTGACATATACAACCGACTTT |
| *clpP* right | GCCTAAAAAAAATAATCTTTCTCGATAAA |
| *atpB* left | CCGAGATGTTAATGAGCAAGAC |
| *atpB* right | CATTCTACCCAATAAGGCGGA |
| *rpoB* left | ATACGAGATATCCATCCTAGTCAC |
| *rpoB* right | GTCCAACATTGATTCCTTCAGAC |
| *rpl23* left | ACTCTTCTTTGGTGTCAAGGT |
| *rpl23* right | GCATTGTATGTCCCAGAATAGGT |
| *accD* left | TTTATGGTTGGGATGAGCGT |
| *accD* right | CCGATATGAAATTGCGAATGTCC |
| *rpoC1* left | TACATAGATTAGGCATACAGTCATTCCA |
| *rpoC1* right | TGCGTCCTTCCACTAAAATAGGTT |
| 18S rRNA | TGCGTCCTTCCACTAAAATAGGTT |
| 18S rRNA | CCCGGAACCCAAAAACTTTG |
